# Supplementary material for: Overexpression of Sedoheptulose-1,7-Bisphosphatase Enhances Photosynthesis in Chlamydomonas reinhardtii and Has No Effect on the Abundance of Other Calvin-Benson Cycle Enzymes
Source: Front Plant Sci. 2020 Jun 23;11:868. doi: 10.3389/fpls.2020.00868 (PMC7324757; doi:10.3389/fpls.2020.00868)
Supplement: Supplementary file 1 [file Data_Sheet_1.PDF]

Supplemental Figure 1

A

```
MASMTGGQQMGRDPAGAKLGGNEQVTRADLNVPLDKTFNDALADAKLSELLGKPVTKAVSLVLPSLKVLIT
      N-terminus     .   iRT   .          PGK                    .    GAP3
APAKALQNTVLKVMFEGILLKSVVSIPHGPSIIAARVPLFIGSKTLLYGGIYGYPGDAKIYSFNEGNYGLW
   .                FBA3              .                  FBP1
DDSVKLTNITGRLLFEALKFLAIDAINKVSTLIGYGSPNKNPDFFNRFIESQVAKGVNPWIEVDGGVTPEN
    .    SBP1     .            TRK1            .           RPE1
AYKSDIIVSPSILSADFSRIYLDISDDIKVAELLDFKGHSLESIKSLFGESNEVVAKLVDELNAGTIPRLA
       RPE1       .          PRK1            .          TPI1          .
NLPEVKLQNIVGVPTSIRTQLSQDELKSGQPAVDLNKASGQPAVDLNKAEAALLVRSNSTPLGSRGILASD
           RPI1           .         CP12           .        RMT1      .
ESNATTGKALQSSTLKVSAADVARALQASVLKVTEAAALASGRNLALELVRSAEGLDASASLRAAWSHHHH
     FBA1         .     FBA2        .    Cre07g338451    .    iRT      .
HHHKAWASWASKLAAALEHHHHHH
         HIS-tag
```

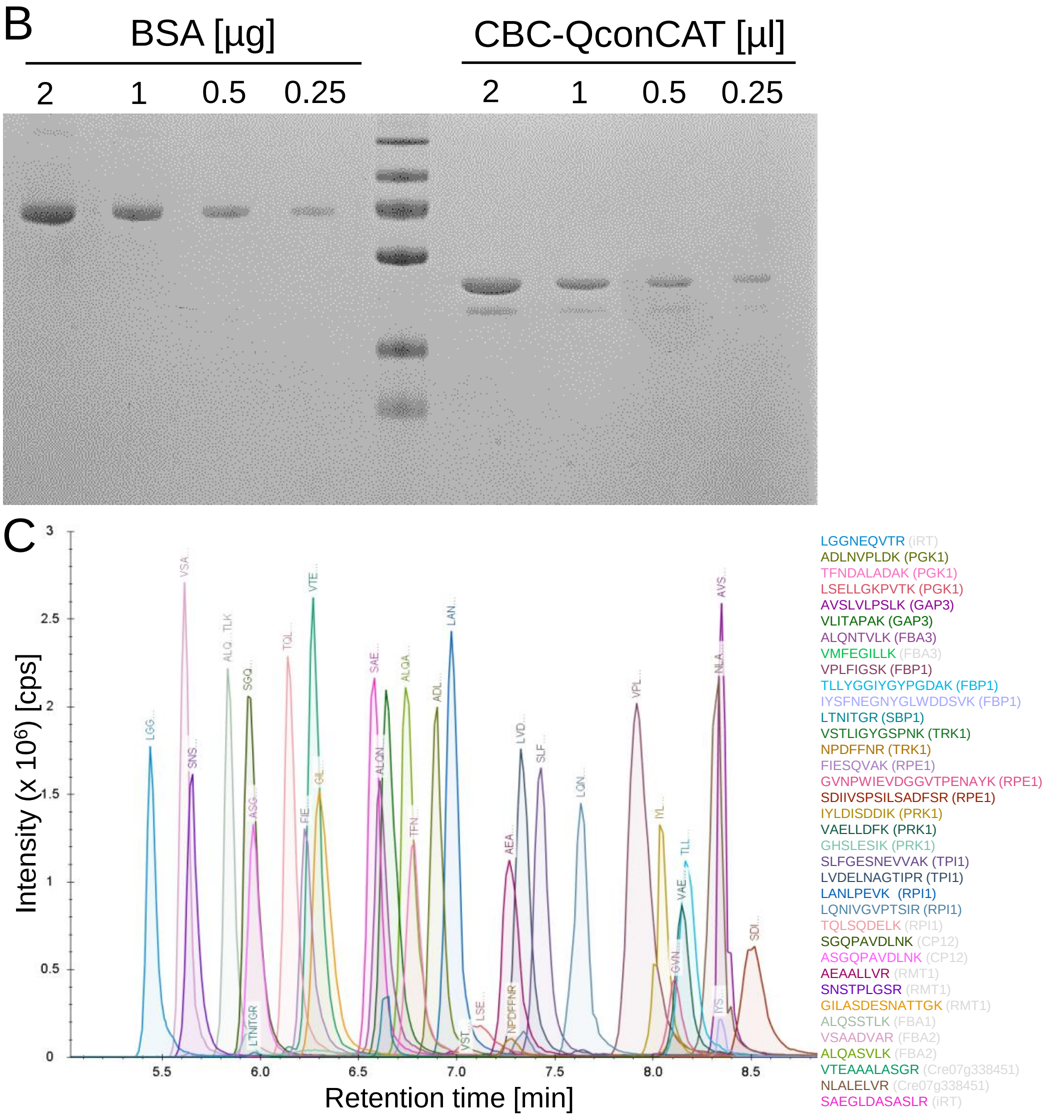

Supplemental Figure 1. Design and production of the Calvin-Benson-Cycle (CBC) QconCAT protein.
(A) Sequence of the CBC-Qprot and protein source of selected Q-peptides. Peptides in grey were erroneously included (CP12), have a proline following the tryptic cleavage site in the native context (FBA3), or belong to a fructose-bisphosphatase not involved in the Calvin-Benson-Cycle (Cre07.g338451). iRT peptides can be used for retention time aligment.
(B) The purified, ^{15}N-labeled CBC-Qprot was quantified on a NanoDrop spectrophotometer and the concentration adjusted to 1 µg/µl. The indicated volumes of the CBC-Qprot were then separated next to a BSA standard on a 12%-SDS polyacrylamide gel and stained with Coomassie blue. The labeling efficiency of the CBC-Qprot was 99.39% ± 0.37%.
(C) Extracted ion chromatograms (XICs) of the proteotypic ^{15}N labeled Q-peptides derived from the PS-Qprot. The purified protein was tryptically digested and run on a short 6-min HPLC gradient. XICs of the resolved peptides were extracted using the PeakView software (ABSciex). Peptides for which the corresponding protein name is given in grey were not used for quantification. Note that due to the very short not all peptides were detected within the retention time window.

Supplemental Figure 2

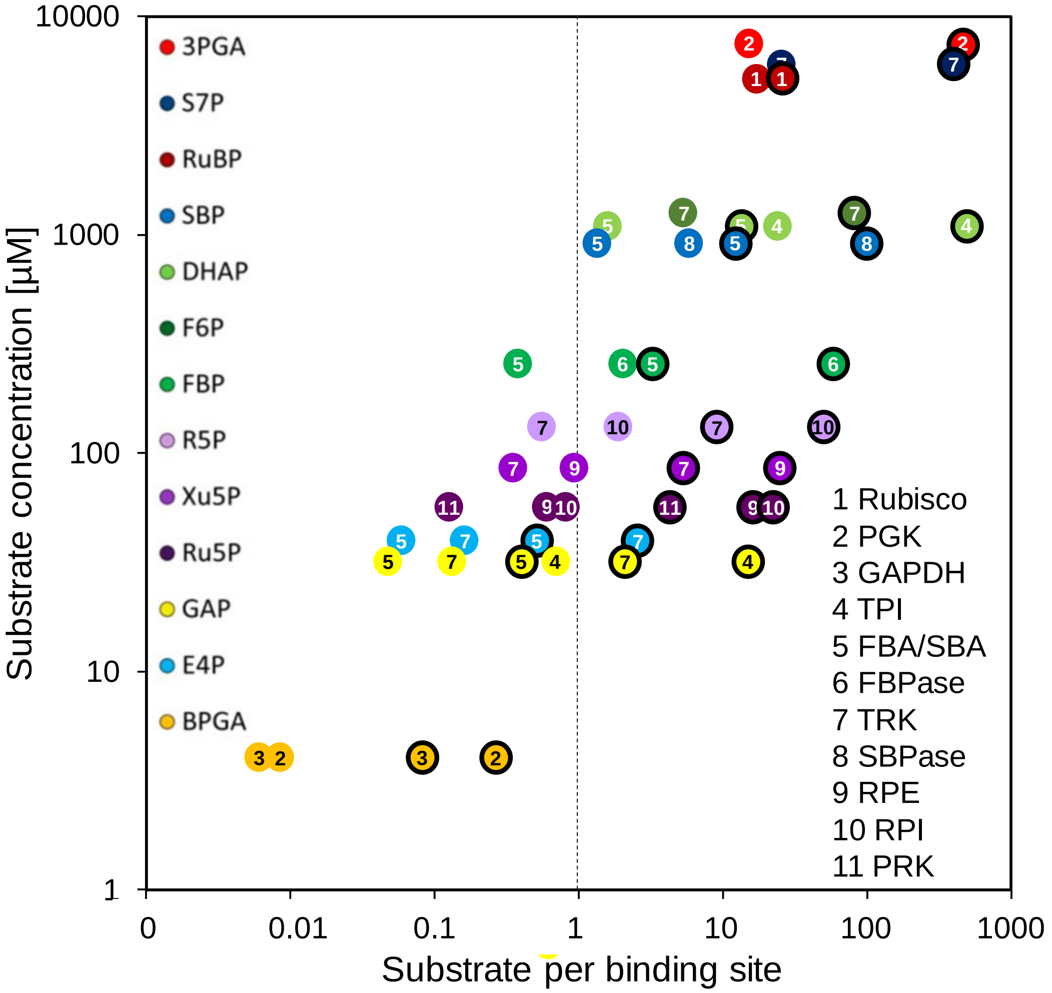

Supplemental Figure 2. Comparison of substrate per binding site versus substrate concentration between Mettler et al (2014) and this study.

Substrate concentrations of CBC reactions, determined by LC-MS/MS in samples taken 20 min after the light intensity was increased from 41 to 145 µmol photons m^{-2} s^{-1}, were taken from Mettler et al. (2014). Binding site abundances of CBC enzymes were determined by the QconCAT approach (Table 2) (circles with black outlines) or the emPAI approach (Mettler et al., 2014) (circles without outline). Substrate per binding site values of CBC reactions are plotted against the substrate concentration. The slightly different values for the Rubisco large and small subunits, as determined in this study, are shown as a mean here.
