# Supplementary material for: Overexpression of Sedoheptulose-1,7-Bisphosphatase Enhances Photosynthesis in Chlamydomonas reinhardtii and Has No Effect on the Abundance of Other Calvin-Benson Cycle Enzymes
Source: Front Plant Sci. 2020 Jun 23;11:868. doi: 10.3389/fpls.2020.00868 (PMC7324757; doi:10.3389/fpls.2020.00868)
Supplement: Supplementary file 2 [file Table_1.DOCX]

CBC-QconCAT: PGK, GAP3, FBA3, FBP1, SBP1, TRK1, RPE1, PRK1, TPI1,RPI1,

CP12, RMT1, FBA1, FBA2

MASMTGGQQMG**R**DPAGA**K**LGGNEQVT**R**ADLNVPLD**K**TFNDALADA**K**LSELLGKPVT**K**AVSLVLPSL**K**VLIT

¯¯¯¯N-terminus¯¯¯.¯¯¯iRT¯¯.¯¯¯¯¯¯¯¯¯¯¯PGK¯¯¯¯¯¯¯¯¯¯¯¯¯¯¯.¯¯¯¯¯GAP3¯¯¯¯¯

APA**K**ALQNTVL**K**VMFEGILL**K**SVVSIPHGPSIIAA**R**VPLFIGS**K**TLLYGGIYGYPGDA**K**IYSFNEGNYGLW

¯¯¯.¯¯¯¯¯¯¯¯¯¯¯¯¯¯FBA3¯¯¯¯¯¯¯¯¯¯¯¯¯.¯¯¯¯¯¯¯¯¯¯¯¯¯¯¯¯¯FBP1¯¯¯¯¯¯¯¯¯¯¯¯¯¯

DDSV**K**LTNITG**R**LLFEAL**K**FLAIDAIN**K**VSTLIGYGSPN**K**NPDFFN**R**FIESQVA**K**GVNPWIEVDGGVTPEN

¯¯¯¯.¯¯¯¯SBP1¯¯¯¯¯.¯¯¯¯¯¯¯¯¯¯¯¯TRK1¯¯¯¯¯¯¯¯¯¯¯.¯¯¯¯¯¯¯¯¯¯¯¯RPE1¯¯¯¯¯¯¯¯

AY**K**SDIIVSPSILSADFS**R**IYLDISDDI**K**VAELLDF**K**GHSLESI**K**SLFGESNEVVA**K**LVDELNAGTIP**R**LA

¯¯¯¯¯¯¯RPE1¯¯¯¯¯¯¯.¯¯¯¯¯¯¯¯¯¯¯PRK1¯¯¯¯¯¯¯¯¯¯.¯¯¯¯¯¯¯¯¯¯¯TPI1¯¯¯¯¯¯¯¯.¯¯

NLPEV**K**LQNIVGVPTSI**R**TQLSQDEL**K**SGQPAVDLN**K**ASGQPAVDLN**K**AEAALLV**R**SNSTPLGS**R**GILASD

¯¯¯¯¯¯¯¯¯¯¯RPI1¯¯¯¯¯¯¯¯¯¯¯.¯¯¯¯¯¯¯¯CP12¯¯¯¯¯¯¯¯.¯¯¯¯¯¯¯RMT1¯¯¯¯¯.¯¯¯¯¯¯

ESNATTG**K**ALQSSTL**K**VSAADVA**R**ALQASVL**K**VTEAAALASG**R**NLALELV**R**SAEGLDASASL**R**AAWSHHHH

¯¯¯¯¯FBA1¯¯¯¯¯¯.¯¯¯¯¯FBA2¯¯¯¯¯¯.¯¯¯Cre07g338451¯¯¯.¯¯¯¯iRT¯¯¯¯.¯¯¯¯¯¯¯¯

HHH**K**AWASWAS**K**LAAALEHHHHHH

¯¯¯¯¯¯¯¯¯HIS-tag¯¯¯¯¯¯¯¯

Proline after lysine in native context

Wrong Q-peptide

Signal peptide determined by Chloro-P; ’/’ indicates experimentally verified cleavage site (Terashima et al., 2010)

Q-peptides used

Q-peptides not used

RPE1 >Cre12.g511900.t1.2 (mature: 26071.21)
MQALQMRSSSKAVGARSAKPSRASAVRVQATSRVDKCKKSDIIVSPSILSADFSRLGDEVRAIDQAGCDWVHIDVMDGRFVPNITIGPLVVEALRPVTDKVLDVHLMIVEPELRIPDFAKAGADIISVHAEQSSTIHLHRTLNMVKDLGCKAGVVLNPGTSLSTIEEVLDVVDLILIMSVNPGFGGQKFIESQVAKIRNLKRMCNEKGVNPWIEVDGGVTPENAYKVIDAGANALVAGSAVFKAKSYRDAIHGIKVSKAPANVMA*

FBA1 >Cre01.g006950.t2.1

MQAKMQTRAVSGRAAARSRVSTTKVVCRALNSMESPYAEELKKTAAYISQKGKGILASDESNATTGKRLESVGVENTEDNRRAWRELLYTAPGLGQYISGAIMFEETLYQKARDGRQFVDILLAQGIYPGIKVDTGLQILPGDKGETTTQGLDGLADRCKAYRKQGARFAKWRAVVKIGEAGCPSTTAVLENAHGLARYAQICQENGLVPIVEPEVTLGPGDYSIEETAFWSERVYSHTMRLLNEYGVVLEGILLKPNMCLPGLDAPVASPQLVAEVTTRTMMRSIPPAVPGIHFLSGGMSEEESTLNLQALNEACPNAPWALTFSYGRALQSSTLKTWAGKESNWAAAQDILLKLAKANSEASTGSFKGPHPVPGGGRILQALRTGGAGK*

FBA2 >Cre02.g093450.t1.2

MASPLAQIAAQLAAPGKGLLASDESTGTIGKRLEKAGLPNTEDIRRSYRELYYTTPGMGQYISGVIMFKETLYQSTKAGRPFVEVLAEQGILAGIKVDEGLEPLAGAADGETHTKGLEGLEANCREYARAGAKFAKWRATLKVTDTLPSALAVERNADELAQYARICQNCGLVPVVEPEILIDGNHSQERFGQVTEQVIGATVAALWRHGVELEGCLLKPQMVIPGADAEGGKVSAADVARCTVAALRRVVPPAIPGIMFLSGGQTEEEATINLDAVNREAQAVGRCPWVLSFSFGRALQASVLKLWSSDQTRVAEAQQLALALARVNSEAALGNYNASSGSHPSTLGTATLHETFRGWNGQPAGNGAAQ*

FBA3 >Cre05.g234550.t1.2 (mature: 38168.59)

MALMMKSSASLKAVSAGRSRRAVVVRA/GKYDEELIKTAGTVASKGRGILAMDESNATCGKRLDSIGVENTEENRRAYRELLVTAPGLGQYISGAILFEETLYQSTASGKKFVDVMKEQNIVPGIKVDKGLVPLSNTNGESWCMGLDGLDKRCAEYYKAGARFAKWRSVVSIPHGPSIIAARDCAYGLARYAAIAQNAGLVPIVEPEVLLDGEHDIDRCLEVQEAIWAETFKYMADNKVMFEGILLKPAMVTPGADCKNKAGPAKVAEYTLKMLRRRVPPAVPGIMFLSGGQSELESTLNLNAMNQSPNPWHVSFSYARALQNTVLKTWQGKPENVQAAQAALLKRAKANSDAQQGKYDATTEGKEAAQGMYEKGYVY*

RMT1 >Cre16.g661350.t1.2

MQQAFRAHAPRRAGGIRGSGSRLKPSVACRAASSVASKAAVESAIAWATKQGAKLEKANLSTDILTDKPILVASADVQPGESLIVVPDAAWVSVPNVAKTTVGKLASSAGLEPWLQLALVLVAERFGSAKSELAGYASSLPEDLGTPLLWSEEETRALAGTQVAGTLNSYLTFFRSTFAQLQAGLFTANPAAFPPAVFTLPNFVWAVAAVRSRSHPPLEGDKIALAPLVDLVSHRRAANTKLSVRSSGLFGRGQVAVVEATRAIRKGEALGMDYAPGKLDGPVLLDYGVMDTASPKPGYSLTLTLDESDKFVDDKADIVEGAGLRPSMTYSITPDQQPGEEMMAFLRLMNIKAMDAFLLESIFRNEVWGFMQEPVSEGNEEAVCAMLAEGARAALAGYPTTLDQDLAALRSNSTPLGSR.AEAALLVRLGEKESLDAVARFFEDRRATQLKRLVYYQERRLRRLGLVDDEGRTTYDNFFKDGIA*

CP12 >Cre08.g380250.t1.2

MQPAASRLIKKFRTVPGDKHTITKMMLTKSVVISRPAVRPVSTRRAVVVRASGQPAVDLNKKVQDAVKEAEDACAKGTSADCAVAWDTVEELSAAVSHKKDAVKADVTLTDPLEAFCKDAPDADECRVYED*

RPI1 >Cre03.g187450.t1.2 (mature: 26365.46)

MMLKASPAARAAARPAARNVRMM/AAPVSTQLSQDELKKQAAWKAVEYVKSGMVVGLGTGSTAAFAVDRIGQLLKEGKLQNIVGVPTSIRTYEQALSLGIPLATLDEQPKLDVAIDGADEVDPNLDVVKGRGGALLREKMVEMASAKFVCIVDDSKLVEGLGGSKLAMPVEIVQFCHKYTLQRLANLPEVKGCEAKLRMNGDKPYVTDNSNYIVDLYFQTPIKDSQAASKAILGLDGVVDHGLFLDMVDVCIIAGATGVTVQERPNPKKH*

TPI1 >Cre01.g029300.t1.2 (mature: 27165.92)

MQLCKVQRASAARSSRASRSQRVEVVCASSAKFFVGGNWKCNGSVANVAKLVDELNAGTIPRGVDVVVAPPFIYIDYVMQHLDRDKYQLSAQNAWIGGNGAFTGEVSAEQLTDFGVPWVILGHSERRSLFGESNEVVAKKTSHALAAGLGVIACIGETLEQRNSGSVFKVLDAQMDALVDEVKDWTKVVLAYEPVWAIGTGVVASPEQAQEVHAYLRQYCAKKLGAAVADKLRIIYGGSVSDTNCKDLSKQEDIDGFLVGGASLKGAAFVTICNAAGPKAKP*

PRK1 >Cre12.g554800.t1.2 (mature: 38567.33)

MAFTMRAPAPRATAQSRVTANRARRSLVVRADKDKTVVIGLAADSGCGKSTFMRRMTSIFGGVPKPPAGGNPDSNTLISDMTTVICLDDYHCLDRNGRKVKGVTALAPEAQNFDLMYNQVKALKEGKSVDKPIYNHVSGLIDAPEKIESPPILVIEGLHPFYDKRVAELLDFK.IYLDISDDIKFAWKIQRDMAERGHSLESIKSSIAARKPDFDAYIDPQKKDADMIIQVLPTQLVPDDKGQYLRVRLIMKEGSKMFDPVYLFDEGSTISWIPCGRKLTCSFPGIKMFYGPDTWYGQEVSVLEMDGQFDKLEELIYVESHLSNTSAKFYGEITQQMLKNSGFPGSNNGTGLFQTIVGLKVREVYERIVKKDVVPV*

TRK1 >Cre02.g080200.t1.2 (mature: 73947.26)

MQTMLKQRCQPAVGKQAKAVPAVAPKVGRARNVVVAQAAPAAAKAAAPSISRDEVEKCINAIRFLAIDAINKSKSGHPGMPMGCAPMGYVLWNEVMKYNPKNPDFFNRDRFVLSAGHGSMFQYSMMHLTGYDSVPLDQIKQFRQWNSLTPGHPENFVTPGVEVTTGPLGQGICNAVGLAVAEAHLAARFNKPDVKPIVDHYTYCILGDGCMMEGISNEACSLAGHWGLGKLIALYDDNKISIDGHTDISFTEDVAKRYEALGWHVIHVINGNTDVDGLRAAIAQAKAVKDKPTLIKVSTLIGYGSPNKADSHDVHGAPLGPDETAATRKNLNWPYGEFEVPQDVYDVFRGAIKRGAEEEANWHKACAEYKAKYPKEWAEFEALTSCKLPENWEAALPHFKPEDKGLATRQHSQTMINALAPALPGLIGGSADLAPSNLTLMKISGDFQKGSYAERNLRFGVREHAMGAICNGIALHKSGLIPYCATFYIFTDYMRNAMRMSALSEAGVVYVMTHDSIGLGEDGPTHQPIEHLASFRAMPDMLMIRPAGGNETAGAYKVAIANRKRPTTIALSRQNMPNIPNCSVEGVAKGAYTIHDTKAGVKPDVILMGTGSELELATAAAGILEKEGKNVRVVSFPCWELFEEQSAEYKESVLPSDVTARVSVEAATSFGWAKYIGLKGKHVGIDTFGASAPAPTLYEKFGITVNHVVEAAKATLQH*

SBP1 >Cre03.g185550.t1.2 (mature: 35980.49)

MMRQKVAGAIAGERRSAVAPKMGRAATAPVVVASANASAFKGAAVTARVKRSTRAARVQSRRTAVLTQAKIGDSLAEFLVEATPDPKLRQLMMSMAEATRTIAHKVRTASCAGTACVNSFGDEQLAVDMVADKLLFEALKYSHVCKLACSEEVPEPVDMGGEGFCVAFDPLDGSSIVDTNFAVGTIFGVWPGDKLTNITGREQVAAGMGIYGPRTVFCIALKDAPGCHEFLLMDDGKWMHVKETTHIGEGKMFAPGNLRATFDNPAYERLINFYLGEKYTLRYTGGMVPDVFQIIVKEKGVFTNVTSPTTKAKLRILFEVAPLALLIEKAGGASSCDGKAVSALDIPILVCDQRTQICYGSIGEVRRFEEYMYGTSPRFSEKVAA*

FBP1 >Cre12.g510650.t1.2 (mature: 38928.17)

MAATMLRSSTQSGIAAKAGRKEAVSVRAVAQPQRQAGAASVFSSSSSGAAARRGVVAQATAVATPAAKPAAKTSQYELFTLTTWLLKEEMKGTIDGELATVISSVSLACKQIASLVNRAGISNLTGVAGNQNVQGEDQKKLDVVSNEVFKNCLASCGRTGVIASEEEDQPVAVEETYSGNYIVVFDPLDGSSNIDAGISVGSIFGIYEPSEECPIDAMDDPQKMMEQCVMNVCQPGSRLKCAGYCLYSSSTIMVLTIGNGVFGFTLDPLVGEFVLTHPNVQIPEVGKIYSFNEGNYGLWDDSVKAYMDSLKDPKKWDGKPYSARYIGSLVGDFHRTLLYGGIYGYPGDAKNKNGKLRLLYECAPMSFIAEQAGGLGSTGQERVLDVNPEKVHQRVPLFIGSKKEVEYLESFTKKH*

GAP3 >Cre01.g010900.t1.2 (mature: 38089.87)

MAAMMQKSAFTGSAVSSKSGVRAKAARAVVDVRAEKKIRVAINGFGRIGRNFLRCWHGRQNTLLDVVAINDSGGVKQASHLLKYDSTLGTFAADVKIVDDSHISVDGKQIKIVSSRDPLQLPWKEMNIDLVIEGTGVFIDKVGAGKHIQAGASKVLITAPAKDKDIPTFVVGVNEGDYKHEYPIISNASCTTNCLAPFVKVLEQKFGIVKGTMTTTHSYTGDQRLLDASHRDLRRARAAALNIVPTTTGAAKAVSLVLPSLKGKLNGIALRVPTPTVSVVDLVVQVEKKTFAEEVNAAFREAANGPMKGVLHVEDAPLVSIDFKCTDQSTSIDASLTMVMGDDMVKVVAWYDNEWGYSQRVVDLAEVTAKKWVA*

PGK1 >Cre11.g467770.t1.1 (mature: 42876.6 kDa)

MALSMKMRANARVVSGRRVAAVAPRVVPFSSASSSVLRSGFAAEVSVDIRRVGRSRIVVEAVKKSVGDLHKADLEGKRVFVRADLNVPLDKATLAITDDTRIRAAVPTLKYLLDNGAKVLLTSHLGRPKGGPEDKYRLTPVVARLSELLGKPVTKVDDCIGPEVEKAVGAMKNGELLLLENVRFYKEEEKNEPEFAKKLAANADLYVNDAFGTAHRAHASTEGVTKFLKPSVAGFLLQKELDYLDGAVSNPKRPFVAIVGGSKVSSKITVIEALMEKCDKIIIGGGMIFTFYKARGLKVGSSLVEDDKIELAKKLEEMAKAKGVQLLLPTDVVVADKFDANANTQTVPITAIPDGWMGLDIGPDSVKTFNDALADAKTVVWNGPMGVFEFPKFANGTVSIANTLAGLTPKGCITIIGGGDSVAAVEQAGVAEKMSHISTGGGASLELLEGKVLPGVAALDEK*
